# High Plasma Levels of Fortilin in Patients with Coronary Artery Disease

**DOI:** 10.3390/ijms23168923

**Published:** 2022-08-10

**Authors:** Masayuki Aoyama, Yoshimi Kishimoto, Emi Saita, Reiko Ohmori, Kojiro Tanimoto, Masato Nakamura, Kazuo Kondo, Yukihiko Momiyama

**Affiliations:** 1 Department of Cardiology, National Hospital Organization Tokyo Medical Center, 2-5-1 Higashigaoka, Meguro-ku, Tokyo 152-8902, Japan; 2 Toho University Graduate School of Medicine, 2-22-36 Ohashi, Meguro-ku, Tokyo 153-8515, Japan; 3 Department of Food Science and Human Nutrition, Faculty of Agriculture, Setsunan University, 45-1 Nagaotouge-cho, Hirakata, Osaka 573-0101, Japan; 4 Research Institute of Environmental Medicine, Nagoya University, Furo-cho, Chikusa-ku, Nagoya 464-8601, Japan; 5 Faculty of Regional Design, Utsunomiya University, 350 Minecho, Tochigi 321-8505, Japan; 6 Faculty of Human Life and Environmental Sciences, Ochanomizu University, 2-1-1 Otsuka, Bunkyo-ku, Tokyo 112-8610, Japan

**Keywords:** apoptosis, atherosclerosis, biomarkers, coronary artery disease, fortilin

## Abstract

Excessive apoptosis is known to be a common feature of atherosclerotic lesions. Fortilin is recognized to have potent antiapoptotic properties. An increased fortilin expression was demonstrated in atherosclerotic lesions, and fortilin knockout mice developed less atherosclerosis. However, no study has reported blood fortilin levels in patients with coronary artery disease (CAD). We investigated plasma fortilin levels in 384 patients undergoing coronary angiography. CAD severity was evaluated as the numbers of stenotic vessels and segments. CAD was found in 208 patients (one-vessel (1VD), n = 86; two-vessel (2VD), n = 68; and three-vessel disease (3VD), n = 53). Plasma C-reactive protein (CRP) levels were higher in patients with CAD than without CAD (median 0.60 vs. 0.45 mg/L, *p* < 0.01). Notably, fortilin levels were higher in patients with CAD than without CAD (75.1 vs. 69.7 pg/mL, *p* < 0.02). A stepwise increase in fortilin was found according to the number of stenotic vessels: 69.7 in CAD(−), 71.1 in 1VD, 75.7 in 2VD, and 84.7 pg/mL in 3VD (*p* < 0.01). Fortilin levels also correlated with the number of stenotic segments (r = 0.16) and CRP levels (r = 0.24) (*p* < 0.01). In a multivariate analysis, fortilin levels were independently associated with 3VD. The odds ratio for 3VD was 1.93 (95%CI = 1.01–3.71) for a high fortilin level (>70.0 pg/mL). Thus, plasma fortilin levels in patients with CAD, especially those with 3VD, were found to be high and to be associated with the severity of CAD.

## 1. Introduction

Fortilin, which is also called the translationally controlled tumor protein, is a 172-amino-acid polypeptide that was originally reported to be abundantly expressed in tumor cells [1,2]. Subsequent studies reported that fortilin was overexpressed in various tumor tissues, including colorectal, lung, and breast cancers, and that its protein levels were positively related to their tumorigenicity [3,4,5,6]. Fortilin came to be recognized as a multi-functional polypeptide that is present in cytosol, nucleus, mitochondria, as well as blood, and been found to play a crucial role in normal physiological function [7,8]. Fortilin was reported to protect cells against apoptosis and to promote cell proliferation [7,8]. Notably, fortilin was shown to have potent antiapoptotic activity in response to various stresses, including oxidative stress [3,5,9], which was also shown to upregulate fortilin levels within cells, thereby protecting against cell death [7]. Moreover, fortilin was reported to have a proinflammatory effect on asthma and skin hypersensitivity and to promote allergic inflammation [10].

Atherosclerotic diseases, such as coronary artery disease (CAD), are considered to be chronic inflammatory diseases [11,12]. The excessive apoptosis of endothelial cells and macrophages and the insufficient clearance of apoptotic cells is known to be a common feature of human atherosclerotic lesions [13,14]. Notably, Pinkaew et al. [2] reported that fortilin expression was increased in human atherosclerotic lesions and that fortilin-deficient mice developed decreased atherosclerotic lesions associated with increased macrophage apoptosis and decreased macrophage infiltration. Furthermore, Cho et al. [15] also demonstrated that the overexpression of fortilin accelerated atherosclerosis in apolipoprotein E knockout mice. These suggested that fortilin may contribute to the progression of atherosclerosis. Recently, fortilin levels in blood were reported to be elevated in patients with malignant tumors, including colorectal and lung cancers [1,4,16], and blood fortilin levels were suggested to be a promising biomarker for apoptosis [8]. However, there has been no study reporting blood fortilin levels in patients with atherosclerotic diseases, including CAD. Therefore, our present study investigated the association of plasma fortilin levels with CAD in 384 consecutive patients undergoing elective coronary angiography for suspected CAD.

## 2. Results

**Figure 1:**
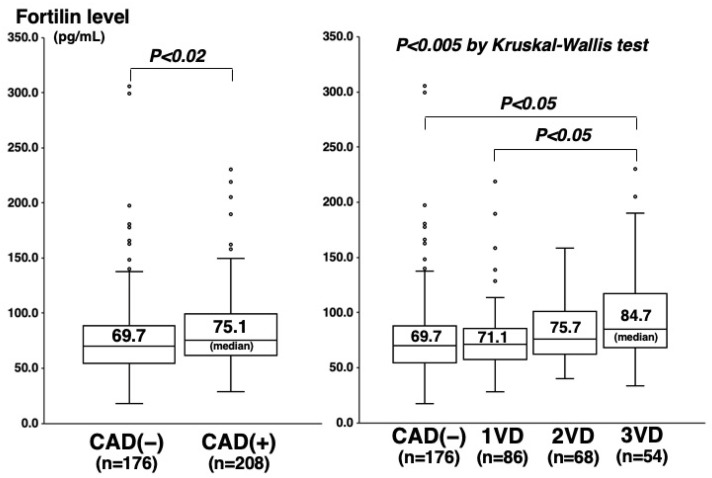
Fortilin levels and CAD or the number of stenotic vessels. Fortilin levels were higher in the CAD(+) group than in the CAD(−) group (*p* < 0.02) (**left**), and they were the highest in the 3VD group (*p* < 0.005) (**right**). The central line and box show the median and 25th to 75th percentiles.

**Figure 2:**
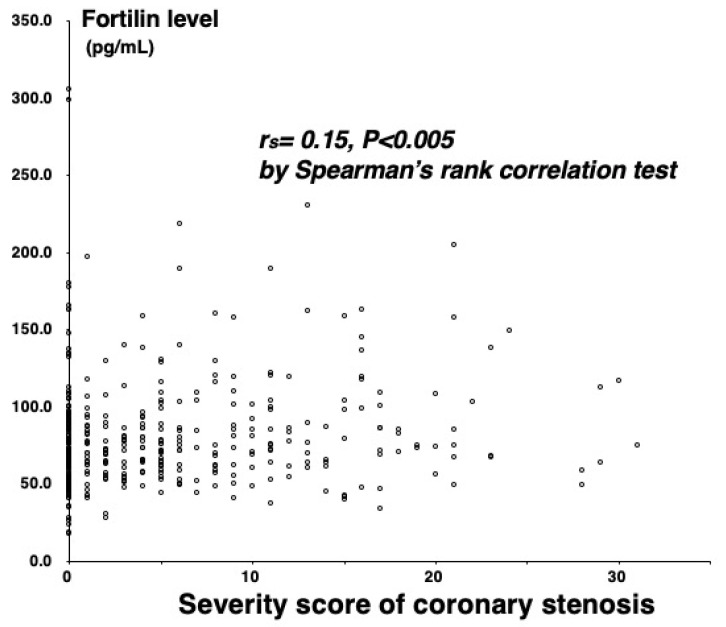
Correlation between fortilin levels and the severity score.

Among the 384 study patients, CAD (>50% stenosis on cine angiograms) was angiographically proven in 208 (54%) (one-vessel (1VD), n = 86; two-vessel (2VD), n = 68; and three-vessel disease (3VD), n = 54). In comparison with the 176 patients without CAD, the 208 with CAD were older and predominantly male, and more often had hypertension, diabetes mellitus (DM), hypercholesterolemia, and low HDL cholesterol levels (Table 1). Plasma C-reactive protein (CRP) levels were also higher in patients with CAD than in those without CAD (median 0.60 vs. 0.45 mg/L, *p* < 0.01) (Table 1). Notably, fortilin levels were higher in patients with CAD than without CAD (75.1 vs. 69.7 pg/mL, *p* < 0.02) and stepwisely increased in the number of stenotic coronary vessels (69.7 pg/mL in CAD(−), 71.1 pg/mL in 1VD, 75.7 pg/mL in 2VD, and 84.7 pg/mL in 3VD), and fortilin levels were the highest in 3VD (*p* < 0.005) (Figure 1). High fortilin levels (>70.0 pg/mL) were found in 49% of patients with CAD(−), 53% of 1VD, 62% of 2VD, and 72% of 3VD (*p* < 0.025) (Table 1). Fortilin levels also correlated with the number of >50% and >25% stenotic segments and the severity score of stenosis (measured using the Spearman’s rank correlation test: r = 0.15; r = 0.16; and r = 0.15, *p* < 0.005) (Figure 2). Furthermore, fortilin levels correlated with CRP (r = 0.24, *p* < 0.001) and HbA1c (r = 0.14, *p* < 0.01) levels, but not with BMI or blood pressure.

**Figure 3:**
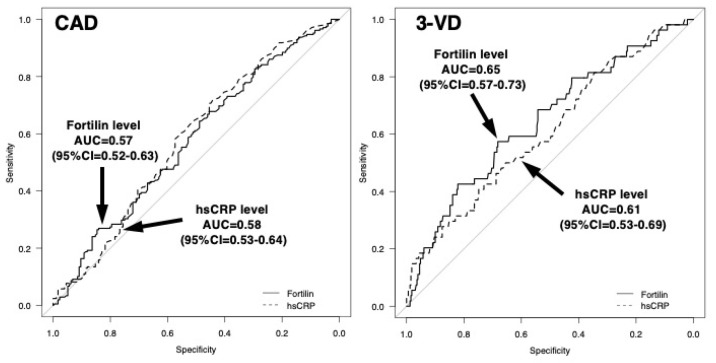
Receiver–operating characteristic (ROC) curves of fortilin and CRP levels for predicting the presence of CAD or 3VD. For the prediction of CAD, the areas under the curves (AUCs) for the fortilin and CRP levels were 0.57 (95%CI = 0.52–0.63) and 0.58 (95%CI = 0.53–0.64). For the prediction of 3VD, the AUC for fortilin was 0.65 (95%CI = 0.57–0.73), which tended to be larger than that for CRP (0.61; 95%CI = 0.53–0.69).

The sensitivity and specificity of the high fortilin level (>70 pg/mL) were 61% and 51% for CAD and 72% and 47% for 3VD, respectively (Table 1). For the prediction of CAD, AUC for fortilin levels was 0.57 (95%CI = 0.52–0.63), which was similar to that for CRP levels (0.58; 95%CI = 0.53–0.64). For the prediction of 3VD, AUC for fortilin was 0.65 (95%CI = 0.57–0.73), which tended to be larger than for CRP (0.61; 95%CI = 0.53–0.69) (Figure 3). To clarify the independent associations of fortilin levels with CAD or 3VD, variables (age, sex, hypertension, hypercholesterolemia, statin, DM, smoking, HDL cholesterol, CRP, and fortilin levels) were entered into a multiple logistic regression model. As a result, fortilin levels were not a significant factor for CAD independent of atherosclerotic risk factors and CRP. However, fortilin levels were found to be an independent factor for 3VD. The odds ratio for 3VD was 1.93 (95%CI = 1.01–3.71) for the high fortilin (>70.0 pg/mL) (*p* < 0.05) (Table 2), but the odds ratios for 1VD and 2VD were 0.83 (0.51–1.36) and 1.20 (0.69–2.08), respectively (*p* = NS).

## 3. Discussion

In the present study, plasma fortilin levels of patients with CAD, particularly those with 3VD, were higher than those without CAD. Moreover, fortilin levels positively correlated with CAD severity. Fortilin did not show a significant association with CAD independent of atherosclerotic risk factors, but it did show an independent association with 3VD.

Regarding the association of fortilin with vascular diseases, pulmonary arterial hypertension (PAH) was recognized to be characterized by the excessive proliferation and impaired apoptosis of pulmonary arterial endothelial cells. An immunostaining study showed fortilin upregulation in the lung tissues of patients with PAH, and fortilin silencing increased apoptosis in blood outgrowth endothelial cells [17]. Similar to patients with cancer [1,4,16], high blood levels of fortilin were reported in patients with idiopathic PAH [18]. These findings, thus, suggested that increased fortilin in PAH, as well as in cancer, leads to an imbalance between cell proliferation/growth and apoptosis and to such disease progression [17,18]. Tulis et al. [19] reported that the adenovirus-mediated gene delivery of fortilin to balloon-injured carotid arteries in rats attenuated neointima thickening with suppressed smooth muscle cell proliferation and apoptosis. However, Pinkaew et al. [2] demonstrated that fortilin knockout mice (fortilin+/−) had less atherosclerotic lesions with an increased apoptosis of macrophages and decreased infiltration of macrophages in intima than fortilin+/+ mice. In human atherosclerotic lesions, the expression of fortilin increased as the degree of atherosclerosis progressed from lesions with a fatty streak to lesions with a fibrous cap [2]. Moreover, Cho et al. [15] also showed that the transgenic overexpression of fortilin exacerbated the progression of atherosclerosis in apolipoprotein E knockout mice [15]. These findings, therefore, suggested that increased fortilin plays a promotive role in the progression of atherosclerosis. On the other hand, fortilin has antiapoptotic and proinflammatory properties [3,10]. However, excessive apoptosis has been recognized to be a common feature of atherosclerotic lesions, and the insufficient clearance of apoptotic cells leads to apoptotic cell accumulation and proinflammatory responses [13,14,20]. Therefore, increased fortilin in atherosclerotic lesions may represent an adaptive response aimed at ameliorating excessive apoptosis.

Fortilin usually exists in cells, but it is recognized to be released into the extra-cellular space as secretory exosomes, and then to be circulating in the blood [1,8]. High blood fortilin levels have been reported in patients with PAH [17,18]. However, there has been no study reporting blood fortilin levels in patients with CAD. For the first time, our study reported that plasma fortilin levels in patients with CAD, particularly those with 3VD, were high and that they were associated with CAD severity. In the multivariate analysis, fortilin levels were not a significant factor for CAD, but they were an independent factor for 3VD. The sensitivity and specificity of a high fortilin level (>70 pg/mL) were 61% and 51% for CAD and 72% and 47% for 3VD, respectively. Our results suggested that fortilin levels reflected the CAD severity and could be a biomarker for CAD, especially for 3VD. However, the correlation of fortilin levels with CAD severity was statistically significant but weak. As shown in Figure 1, there was a substantial overlap in fortilin levels between the CAD(−) and CAD(+) patients. Since we previously reported that CRP levels were more closely correlated with the degree of aortic atherosclerosis than that of coronary atherosclerosis [21], fortilin levels in patients with CAD may reflect not only coronary atherosclerosis, but also atherosclerosis in other vascular beds. A further study is necessary to clarify the major source and role of high levels of fortilin in the blood of patients with CAD.

In vitro, fortilin was reported to protect pancreatic β-cells from apoptosis [22]. Transgenic mice with the overexpression of fortilin showed improved glucose tolerance and insulin sensitivity [23]. Moreover, transgenic mice with an overexpression of fortilin also developed hypertension associated with an increase in contractility and a decrease in the relaxation of vascular smooth muscle cells through the inhibition of Na,K-ATPase activity [8,24]. These factors, thus, suggested that fortilin may contribute to the development of DM as well as hypertension. In our study, fortilin levels significantly correlated with HbA1c (r = 0.14), but not with BMI or blood pressure. However, fortilin levels were found to be associated with 3VD independent of DM and hypertension, as well as BMI.

Fortilin is considered to be a potential target for cancer therapy. Recombinant fortilin was reported to promote invasiveness of cancer cells in vitro and metastasis in a mouse model [16]. Levomepromazine and buclizine therapy was shown to reduce fortilin expression in cancer cells and to inhibit their cell growth [18]. Moreover, sertraline and thioridazine therapy neutralized fortilin and led to the apoptosis of cancer cells [6]. Because of the atherogenic effects of fortilin, antifortilin therapy was suggested to be a promising antiatherosclerotic therapy [2,15]. However, since increased fortilin in atherosclerotic lesions may be an adaptive response to ameliorate excessive apoptosis, further studies are necessary to clarify the role and mechanism of increased fortilin in atherosclerotic diseases, including CAD.

The present study was associated with some study limitations. First, the degree of coronary atherosclerosis was assessed with angiography. It could not look at plaques and only visualized the characteristics of lumens in stenotic lesions. However, intravascular ultrasound was not always performed. Second, we did not assess fortilin levels in the blood of coronary sinus. We could not provide any information about the main sources of fortilin in plasma. Third, excessive apoptosis is recognized to be related to coronary plaque instability, leading to the development of acute coronary events, such as acute coronary syndrome (ACS) [13,14]. However, our study did not include any patient with acute coronary events. The further study of patients with ACS is necessary to clarify the potential role and diagnostic ability of fortilin in ACS. Moreover, to elucidate the prognostic value of fortilin levels for future coronary events, a prospective study is also needed. Fourth, because our study was a cross-sectional one, it could not establish causality, but only showed some hypotheses as well as some associations. Fifth, our study was carried out in patients who had an angiography, and such patients were generally a highly selected population. Our results may not be applied to the general population. Moreover, Khan et al. [25] reported that obesity is related to the higher incidence of cardiovascular events. However, in our study, no significant difference was found in BMI between the Japanese CAD(−) and CAD(+) patients. In the report by Fumisawa et al. [26], there was no difference in BMI between Japanese CAD patients and age- and sex-matched controls. Saito et al. [27] also reported that obesity was not an independent factor for CAD among 38,385 Japanese patients without prior CAD. Therefore, our results may not be applicable to other ethnic populations. Finally, no healthy controls were included in our study. We compared CAD(−) patients and CAD(+) patients. Even CAD(−) patients can present with some degree of atherosclerosis in their coronary arteries and in other vascular beds.

## 4. Materials and Methods

### 4.1. Study Patients

In 2008, our study prospectively started collecting blood samples as well as angiographic data of patients undergoing coronary angiography at the NHO Tokyo Medical Center. The institutional review board approved our study (reg. no. R08-050/R21-037). Our study was performed according to the Declaration of Helsinki. After obtaining written informed consent, blood sampling was undertaken after overnight fasting on the day when angiography was scheduled. In the present study, we assessed plasma fortilin levels in 384 consecutive patients who had coronary angiography. Excluded were any patients with ACS, such as acute myocardial infarction and class III unstable angina [28]. Additionally, excluded were patients with any history of coronary artery bypass surgery or percutaneous coronary intervention, or those with heart failure, severe valvular heart disease, or aortic disease. Moreover, since blood fortilin levels have been shown to be elevated in patients with malignant tumors [1,4,16], any cancer patients were excluded. We defined hypertension as blood pressure (BP) of ≥140/90 mmHg and/or the presence of drug prescriptions; 222 patients (58%) were on antihypertensive medication. We also defined hypercholesterolemia as having an LDL cholesterol of >140 mg/dL and/or the presence of drug prescriptions, and 150 patients (39%) were on statin. DM was measured using fasting glucose levels ≥126 mg/dL and/or the presence of drug prescriptions or insulin treatment, and 104 patients (27%) had DM. Smoking was defined as ≥10 packs per year, and 163 patients (42%) were smokers.

### 4.2. The Measurements of Plasma Fortilin and CRP Levels

Blood sampling was undertaken using tubes with EDTA, and then the plasma was frozen until use at −80 °C. For the measurement of fortilin levels, enzyme-linked immunosorbent assay (ELISA) (Human Translationally controlled tumor protein (TPT1) ELISA kit, CUSABIO, Wuhan, China) was used. According to the manufacturer’s data, the measuring range was 12.5 to 800 pg/mL. The intra-assay and inter-assay coefficients of variation were <8% and <10%. To measure high-sensitivity CRP levels, a BNII nephelometer (Dade Behring, Tokyo, Japan) was used.

### 4.3. The Assessment of Coronary Angiography

Angiography was performed with the Philips Electronics cine angiogram system. All the cine angiograms were assessed by one cardiologist, blind to the patients’ data. We defined CAD as at least one coronary artery having >50% luminal diameter stenosis. Moreover, the CAD severity was evaluated as the number of >50% stenotic vessels and >50% and >25% stenotic segments and the severity score of stenosis. We scored the degree of stenosis in each segment from 0 to 4 points (0 = 25% or less; 1 = 26–50%; 2 = 51–75%; 3 = 76–90%; 4 = more than 90% stenosis), and the coronary artery was divided into 29 segments using the CASS classification. We defined the severity score as the sum of the scores of all 29 coronary segments.

### 4.4. Statistics

We statistically analyzed our data using the IBM SPSS software package (ver. 25) and defined statistical significance as a *p*-value of <0.05. Continuous variables are shown as mean ± SD, and categorical variables are shown as number and proportion (%). Since the measured fortilin and CRP levels were not normally distributed and were recognized as nonparametric, the results were shown as median value and interquartile range. For parametric variables, an unpaired *t*-test was used for differences between 2 groups, and ANOVA with Scheffe’s test was used for those among 3 or more groups. For nonparametric variables, the Mann–Whitney U test was used for differences between 2 groups, and the Kruskal–Wallis test with the Steel–Dwass test were used for those among 3 or more groups. For categorical variables, the χ^2^ test was used. The correlations of fortilin levels with CRP levels or CAD severity were assessed with Spearman’s rank correlation test. By creating a ROC curve, we determined the optimal cut-off point of fortilin for CAD to be 70.0 pg/mL with the highest Youden index. Regarding CRP, we used the previously reported cut-off point of 1.0 mg/L for CAD [29,30]. A multiple logistic regression analysis was performed to show the independent association of fortilin levels with CAD.

## 5. Conclusions

Plasma fortilin levels in patients with CAD, particularly those with 3VD, were found to be high and to be associated with CAD severity. Fortilin levels were a significant factor associated with 3VD. Our results suggested that fortilin contributes to the progression of coronary atherosclerosis.

## Figures and Tables

**Table 1:** The clinical characteristics of the study patients.

	CAD(−) (n = 176)	*p*-Value CAD(−) vs. CAD(+)	CAD(+) (n = 208)	1VD (n = 86)	2VD (n = 68)	3VD (n = 54)	among 4 Groups
Age (yrs)	64 ± 13	<0.001	70 ± 10	69 ± 10	70 ± 10	72 ± 9	<0.001
Sex (mem)	103 (59%)	0.001	156 (75%)	65 (76%)	52 (76%)	39 (72%)	0.007
BMI (kg/m^2^)	25.4 ± 13.2	0.139	24.0 ± 3.5	24.3 ± 4.0	24.1 ± 2.8	23.4 ± 3.7	0.467
Hypertension	103 (59%)	<0.001	163 (78%)	68 (79%)	49 (72%)	46 (85%)	<0.001
Systolic BP (mmHg)	131 ± 20	0.083	134 ± 20	133 ± 18	137 ± 20	134 ± 22	0.202
DM	30 (17%)	<0.001	74 (36%)	24 (28%)	26 (38%)	24 (44%)	<0.001
HbA1c (%)	6.0 ± 0.8	<0.001	6.3 ± 1.0	6.2 ± 0.9	6.3 ± 1.0	6.5 ± 1.0	0.001
Smokers	61 (35%)	0.005	102 (49%)	45 (52%)	33 (49%)	24 (44%)	0.030
Hypercholesterolemia	74 (42%)	<0.001	127 (61%)	54 (63%)	41 (60%)	32 (59%)	0.003
Statin	48 (27%)	<0.001	102 (49%)	45 (52%)	32 (47%)	25 (46%)	<0.001
LDL cholesterol (mg/dL)	113 ± 27	0.563	112 ± 30	107 ± 26	112 ± 32	118 ± 33	0.173
HDL cholesterol (mg/dL)	59 ± 17	<0.001	52 ± 13	53 ± 12	52 ± 12	51 ± 15	<0.001
C-reactive protein	0.45	0.005	0.60	0.56	0.67	0.75	0.004
(mg/L)	[0.22, 1.03]		[0.31, 1.32]	[0.25, 0.97]	[0.39, 1.31]	[0.38, 2.31]	
>1.0 mg/L	46 (26%)	0.310	65 (31%)	21 (24%)	22 (32%)	22 (41%)	0.146
Fortilin (pg/mL)	69.7	0.013	75.1	71.1	75.7	84.7	0.002
	[54.4, 87.6]		[61.6, 98.7]	[57.3, 85.2]	[62.0, 100.0]	[68.1, 115.8]	
>70.0 pg/mL	87 (49%)	0.024	127 (61%)	46 (53%)	42 (62%)	39 (72%)	0.017

The data show mean ± SD or number (%), apart from CRP and fortilin, which are shown as median and interquartile range.

**Table 2:** Factors for CAD and 3VD.

	Odds Ratio	(95%CI)	*p*-Value
**CAD**			
Age (10-year increase)	1.74	(1.40–2.16)	<0.001
Sex (male)	2.32	(1.35–4.01)	0.002
Hypertension	1.80	(1.08–2.97)	0.023
Hypercholesterolemia	2.52	(1.57–4.04)	<0.001
Low HDL cholesterol (<40 mg/dL)	2.17	(1.06–4.45)	0.034
Smoking	1.71	(1.04–2.81)	0.035
**3VD**			
Age (10-year increase)	1.52	(1.11–2.07)	0.009
DM	2.29	(1.25–4.20)	0.007
High fortilin (>70.0 pg/mL)	1.93	(1.01–3.71)	0.047

The dependent variables were CAD and three-vessel disease. Analysis included age, sex, BMI, hypertension, hypercholesterolemia, statin, DM, smoking, HDL cholesterol, CRP, and fortilin (>70.0 pg/mL) levels.

## Data Availability

The data that support the findings of this study are available from the corresponding author on reasonable request.
